# Is the effect of a health crisis symmetric for physical and digital financial assets? An assessment of gold and bitcoin during the pandemic

**DOI:** 10.1371/journal.pone.0288762

**Published:** 2023-11-14

**Authors:** Waqar Badshah, Mohammed Musah, Asad Ul Islam Khan

**Affiliations:** 1 Department of Management Information System, Istanbul University, Istanbul, Turkey; 2 Department of Economics, Ibn Haldun University, Istanbul, Turkey; Usak University: Usak Universitesi, TURKEY

## Abstract

The emergence of the covid-19 health crisis, in this advanced technological era where connections between markets, nations, and economies have grown stronger than ever before, the shock of the COVID-19 pandemic quickly had an impact on both physical and digital financial assets. The Chinese financial market experienced the first consequences of the covid-19 pandemic, then spilled over to other financial markets, including those for cryptocurrencies and the precious metals. This study examines the impact of the covid-19 pandemic on the volatilities of the dynamics of bitcoin and gold. Both assets share some characteristics, such as online trading platforms, however, gold is a tangible financial asset unlike bitcoin, which is digitally generated without any physical form. This study argues that the similarities and differences between bitcoin and gold play major roles in how the covid-19 crisis affected their respective dynamics. Using daily data ranging from 9/22/2014 to 1/31/2023 and employing ARMA as the mean equation for GARCH model, the impact of the health crisis (covid-19) is examined on the volatilities of the prices and volumes of bitcoin and gold. Empirical evidence points out that, the pandemic has a symmetric impact on the volatilities of bitcoin and gold price returns, causing them to be more volatile. The impact of the covid-19 observed on the volume returns of the assets, however, is asymmetrical. The empirical results give evidence to the role that the vital differences existing between these assets played during the covid-19 pandemic.

## 1. Introduction

In the world of technological advancement, the connections between international markets, financial systems, and nations’ monetary policies are now stronger. This has resulted in severe spillovers from one economy to another, from one financial market to another, as well as from one international financial asset to another. Portfolio risks are increasing as asset diversification, a method of reducing risks, loses effectiveness due to the integrations and spillovers across assets being more severe than before. Higher fluctuations in prices and volumes of financial assets are typically observed when the financial markets are in crisis. These financial crises are usually fueled by environmental uncertainties, speculations, bad news, inefficiency in the macroeconomic variables, or even epidemics. The World Health Organization (WHO) classified the coronavirus (covid-19), which first appeared in China in December 2019 and later expanded to other regions of the world, to be a pandemic on March 11, 2020. The Chinese financial market experienced the first consequences of the covid-19 pandemic. Due to the current technological advancement, the connections between China’s main stock markets and other financial markets like the cryptocurrency and precious metals markets are tighter than before. The volatility connections between these markets developed much stronger during the covid-19 health crisis. Similar to how the COVID-19 virus propagated throughout the world, the shocks in the Chinese stock markets have an adverse effect on other global financial markets, making financial assets like bitcoin, gold, and others more volatile and riskier during the pandemic [1, 2].

The financial aspects of the global economy have undergone significant change as a result of the rapid development and rise of technology in the latter half of the 20th century. Notable innovations include the use of credit and debit cards, automated teller machines (ATM), Internet banking and other systems that all aim to address the liquidity, supply, and circulation of money in some ways. On October 31, 2008, the long-awaited possibility of opening doors to future currencies emerged: Bitcoin. A cryptocurrency, Bitcoin, was introduced on October 31, 2008, for the first time with a computer science paper explaining how it would function. Moreover, the code was released on January 3, 2009and the first bitcoins popped up [3]. A cryptocurrency is a virtual or digital currency that uses a cryptographic system to secure legitimate transactions with a decentralized structure, which frees them from being under the control of a centralized authority but is rather based on a distributed network across a wide range of computers. This enables direct virtual payment between two users without the involvement of any centralized, trusted authorities like banks or governments.

Fast, secure, cheaper, and more comfortable means of transactions across the globe are some of the pros they come with. They also have the feature of keeping the information of parties in the transaction private and anonymous enough, which makes it an easier means of payment for some illegal economic activities and underground markets. Bitcoin is the first cryptocurrency and is currently the most dominant in the cryptocurrency market. The focus of academic economic research has turned to the cryptocurrency bitcoin, among others, especially because of its amazing blockchain technology. Bitcoin is a computer-based currency not backed by any legal currency and has no physical form. Therefore, it offers no guarantee, which makes its volatilities higher in nature than other financial assets like gold [4, 5]. The development of bitcoin and its extreme volatility, which is grabbing the attention of the entire globe, have opened a whole new set of questions.

Historically, one of the earliest forms of money is regarded to be gold [6, 7]. Among the precious metals market, Gold has been the most dominant since history began, and it is still the most recognized trusted precious metal internationally. It has the reputation of serving as a strong asset to store value and a medium of exchange for centuries due to its excellent physical characteristics, durability, portability, divisibility, and unique standardized features [8]. Throughout history, Gold has been kept for being precious. This is due to its scarcity and limited supply. The free market determines gold prices, that is, the forces of demand and supply, after the end of the Bretton Woods System in 1971. Since then, the prices of Gold have become more volatile than before. Gold is also argued to be negatively correlated with financial cycles and provides shade during a financial crisis [9]. Evidence from [10], due to the low responsiveness of gold supply to price changes, the price of Gold in the short run is mainly determined by the forces coming from demand. In the long run, both the forces of demand and supply set the price of Gold, monetary macroeconomic instruments and political activity indicators mostly have great effects on the price of Gold.

Gold has online platforms as well as bitcoin, where both assets can be traded online worldwide. As of the beginning of February 2023, the market capitalization in the precious metals is led by Gold with a market capitalization of around 12 Trillion US dollars at the price of 1,900 US dollars per ounce, followed by silver and palladium with 1.2 Trillion and 250 Billion respectively. In the market of cryptocurrencies, there are more than 11,000 cryptocurrencies in the world, and the total market capitalization of all cryptocurrencies was around 1.1 trillion US dollars. The market capitalization of the leader, Bitcoin, was 450 billion, followed by Ethereum with 204 billion at prices around 23,500 and 1,668 US dollars, respectively (source: Yahoo Finance).

Bitcoin and Gold are traded online worldwide, but Gold can also be traded in person due to its physical form, is mined physically, and is also used in many physical ways, whereas bitcoin has no physical form as it is digitally generated and cannot be used in any physical form. According to several studies, there are several noteworthy characteristics between bitcoin and gold, both assets are believed to have apolitical attributes and safe haven features during a crisis and are not dependent on inflation for their price increase [9]. Due to these relevant properties that bitcoin possesses as Gold, it has gained attributes like "the digital gold “[11, 12]. In some aspects, bitcoin tends to have a unique upper hand because bitcoin purely relies on a cryptographic system on computers which is independent of decisions and politics of the country’s authorities. Therefore, bitcoin is not affected by the general trend and financing of common assets like Gold. These common characteristics and differences between bitcoin and Gold make it reasonable and relevant to investigate the volatilities of their returns during times of stress like the period of covid-19 pandemic. We investigate the role their similarities and differences played during the period of the pandemic, that’s, period where governments and authorities all over the world implemented many fiscal policies and restricted both some physical and economic movements trying to minimize the stress and impact of the covid-19 pandemic on both the physical and economic health of the world. From [13, 14] studies, both the physical financial and digital financial markets were affected by the shock of the covid-19 pandemic.

Hence this study investigates the impact of the covid-19 health crisis on the volatility returns of prices and volumes of bitcoin and Gold. This paper argues that these similarities and differences between these financial assets play major roles in how the covid-19 crisis affected their respective dynamics. The generalized autoregressive conditional heteroscedastic (GARCH) models have proved to be strong and consistent in modelling time series in which their volatilities vary with time. Bitcoin and Gold both have time-varying volatilities and are also strongly dependent on their past values. Therefore, this study investigates the volatilities of bitcoin prices and volumes and gold prices and volumes using the autoregressive moving average (ARMA) as the mean equation in the standard GARCH to check the impact of the covid-19 on the volatilities in both the mean and variance of the dynamics of bitcoin and Gold. We found that, the covid 19 pandemic had similar impact on the fluctuations of the prices of both bitcoin and gold, however different impacts were recorded on the volumes of bitcoin and gold. This paper is divided into five parts. The introduction is the first chapter, followed by the related literature review, the third chapter describes the data and methodology employed in the study. The estimation results and empirical analysis are in the fourth chapter, and the final chapter is the conclusion.

## 2. Literature review

Recently, in cryptocurrencies markets, bitcoin, and precious markets, Gold have been on top of the most popular and important economic fields driving the world’s economy and resulting in many discussions and grabbing the attention of many researchers. Wide range of literature on bitcoin and Gold from different aspects such as their price dynamics, internal and external shocks to the markets, and how they perform under the shocks. After the emergence of the health crisis, covid-19, several pieces of literature have been released trying to examine the effects of this health crisis on all bitcoin and Gold dynamics.

Bitcoin is already a computer-based currency and is not backed by any legal currency and has no physical form; therefore, it does not give any sort of guarantee, which makes its volatilities higher in nature than other financial instruments like Gold [4, 5]. The pandemic disease, Covid-19, emerged in China and started to spread to other parts of the world in 2019. The Chinese financial market experienced the first consequences of the covid-19 pandemic, and the volatility connections between China’s main stock markets and bitcoin developed strongly during the health crisis, the shocks in the Chinese stock markets spillover to the other financial markets in the world causing financial assets like bitcoin, Gold, and others to be more volatile and riskier during the period of the pandemic [1, 2]. Also, [15] employed an hourly dataset for cryptocurrencies from September 2019–September 2020 and argued that an increase in the volatilities of the returns of cryptocurrencies was observed after the announcement of the covid-19 pandemic compared to the periods before the declaration of the covid-19 as a pandemic.

Similarly, [16] researched the influence fear sentiments due to the pandemic have on the price dynamics of Bitcoin. They used the hourly Google search data about the covid-19 as a proxy, and their results indicate that the volatility of the Bitcoin market intensively became worse as searches about the covid-19 increased. This led to negative price returns for Bitcoin and an increase in the trading volume of Bitcoin.

The returns of Gold became negative after the spread of covid-19 worldwide, yet it provided a safe haven during the world financial crisis caused by the health crisis covid-19. Historically, Gold has a reputation of being classified as a "safe haven" as it has been seen as a natural currency and a very strong value metal. It is also negatively correlated with financial cycles, which means it provides shade during financial crises [9, 17]. As the supply of Gold was hugely affected by the covid-19, while it could provide a safe haven, the demand increased, resulting in the rise of its prices during the period of the pandemic. Evidence from [10] reveals that due to the low responsiveness of gold supply to price changes, the price of Gold in the short run is mainly determined by the forces coming from demand. In the long run, both the forces of demand and supply set the price of Gold. Monetary macroeconomic instruments and political activity indicators mostly have great effects on the price of Gold.

Moreover, [18] employed a daily dataset covering the period from January 2019 to June 2021 using TVP-VAR and provided results that, during the Covid-19 pandemic, Gold was able to maintain its safe haven role in the financial markets while Bitcoin rejected the safe haven role. Their results also show that the role gold played as a safe haven in the financial markets increased and became stronger when the covid-19 disease rapidly spread, but [19] argued for both bitcoin and Gold where they claim the volatility of bitcoin prices became very intense due to the covid-19 pandemic; however, bitcoin can maintain its safe haven role despite the intensified volatility caused by the covid-19. Moreover, bitcoin responses to the covid-19 events are not immediate; the shocks take time before reflecting in the bitcoin markets.

However, [20] employed the multivariate asymmetric dynamic conditional correlation model and argued that the feature of Gold and bitcoin as hedging assets in minimizing the risk in worldwide portfolios is statistically significantly reduced due to the covid-19. Moreover, the safe-haven property of Gold has become weak, and the higher volatility of Bitcoin makes it unable to supply shelter as a safe haven during the pandemic. During the covid-19 pandemic, bitcoin prices statistically significantly became highly volatile. Moreover, the volatility correlation between the bitcoin prices and bitcoin’s trading volume is positive [21] hey employed the ARMA GARCH model with time series data set of bitcoin from January 2012 to April 2020. Their empirical results also point to a significant positive connection between news flow and condition volatility as well as a significant inverse connection between information asymmetry and the price returns of bitcoin. Moreover, [22] investigated the impacts of the covid-19 on the volatilities of several financial variables, including bitcoin prices and gold prices, by employing the ARMA-EGARCH model using a daily data set from September 2019 to December 2020. They provided evidence that within that short period, the covid-19 pandemic had a statistically significant impact on the bitcoin and gold prices, causing excessive fluctuations in their prices and contaminating mutual volatilities between them.

[23] employed the NARDL model using a dataset from the early stages of the pandemic (March 2020–June 2020). Their empirical results show that the correlation between cryptocurrencies and Gold increased, and more of the cryptocurrencies had their returns cointegrated with the returns of Gold. They also provided evidence that during the covid-19, cryptocurrencies formed both short- and long-term asymmetric responses to the returns of Gold, especially to the negative changes in the returns of Gold.

## 3. Data and methodology

The source of the data and the econometric methodologies employed in this study is detailed in this section of this paper.

### 3.1 Data sources

The data sample used in the study is presented in the Table 1 below as well as the sources, the time period and the abbreviations used in representing the various variables in the study.

**Table 1:** Variables and data sources.

Variable(s)	Representations	Data frequency	Time period	Data source
**Bitcoin Price**	BP	Daily	9/22/2014–1/31/2023	Yahoo Finance
**Bitcoin Volume**	BV	Daily	9/22/2014–1/31/2023	Yahoo Finance
**Gold Price**	GP	Daily	9/22/2014–1/31/2023	Yahoo Finance
**Gold Volume**	GV	Daily	9/22/2014–1/31/2023	Yahoo Finance

The original values of the prices and volumes of the variables in used were then to returns series form for the sake of further better and simpler econometric analysis. The returns of the series are employed for the estimations in this paper, many remarkable financial researches make use of returns of the series as it gives easy and complete info to investors about the risks and opportunities of the assets, for statistical purposes, the returns are simpler and effective to employ than the actual prices of the assets due to its unique statistical features [24, 25].

The mathematical expression of the returns of series is expressed as;

rt=ln(1+Rt)=ln(XtXt−1)=xt−xt−1

$$p_{t}=\ln(X_{t})$$

$R_{t}=\sum_{i=1}^{N}w_{i}R_{it},$ *R*_*it*_ = Simple Return of series *i*

$$r_{t}\approx \sum_{i=1}^{N}w_{i}r_{it}$$

Where; *r* = the return at time “t”, *x* = the series value at time *t*.

### 3.2 Methods

This paper employs various econometric methodologies, starting from checking the stationarity of the time series variables in study and examines the impact the health crisis Covid-19 has on the volatilities of the price returns and volumes returns of bitcoin and gold by employing the GARCH model, using ARMA as the mean equation

### 3.3 Unit root test

The unit root test is a common method for testing stationarity in a time series in determining whether a variable follows a random walk or not. Using a set of data for estimations and analyses which isn’t stationary may give misleading and spurious results as data sets having unit root turns to be bias which may lead to a biased result when used. It is then necessary to make check for the stationarity of a data set before using it in estimations.

In testing for the stationarity of the variables used in the study, three different unit root testing methods were employed, the Dickey-Fuller GLS (DF GLS), the Elliott-Rothenberg-Stock Point-Optimal (ELRS Optimal) and the Kwiatkowski-Philips-Schmidt-Shin (KPSS). The first unit root estimation method employed is the Dicky-Fuller GLS (DF GLS), the DF-GLS is a modified and improved method of the Augmented Dicky–Fuller (ADF) for testing unit root in a time series by transforming the time series using the generalized least squares (GLS) before performing the test. The DF-GLS was proposed by Elliot, Rothenberg and Stock (ERS) in their 1996 Econometrica article. The DF-GLS has significantly improved power when an unknown mean or trend is present in the series as compared to the previous versions of the ADF. Trend is included in the DF-GLS test by default therefore the test can be run under two specifications, with trend and intercept or with trend only, the null hypothesis of the DF-GLS is similar to the ADF thus the series is a non-stationary. The null hypothesis of non-stationary is rejected when the T-statistic is less than the critical values of the DF-GLS test. The Elliott-Rothenberg-Stock Point-Optimal (ERS Optimal) is another relevant methodology used in assessing the stationarities of the respective time series under study. The Elliot, Rothenberg & Stock 1996 proposed a point optimal “P-test” which considers the serial correlation which may be present in the error term in order to improve the power of the unit root test. The null hypothesis of the ERS point optimal is non-stationary against alternative of stationary of the observable time series. The null hypothesis of non-stationary is rejected when the P-statistic is less than the critical values in using the ERS optimal tests. The other methodology for checking the stationarities of the variables is the Kwiatkowski-Philips-Schmidt-Shin (KPSS), the KPSS tests are used on the null hypothesis that the time series is stationary against alternative of unit root, that is opposite to most of the various unit root tests in the literature such as Dickey-Fuller, DF-GLS and Phillips-Perron tests. Therefore, the KPSS tests are commonly used as complementary tests to verify the results of the other unit root tests. The KPSS uses a one-sided LM statistic, therefore if the LM test statistic is greater than the critical value the null hypothesis of stationary is rejected meaning the time series variable under observation is non-stationary.

### 3.4 Specification of the conditional mean equation

The ARMA model is used as the mean equation of the GARCH model used in this study, the ARMA is a commonly used statistical method in models for analysis to get the predictions for time series as the ARMA uses the lag order process of the time series taking into accounts the past shocks and values of the time series in prediction of the future values.

The ARMA combines the interactions of the lag orders of the Autoregressive (AR) and the Moving Average (MA) of the time series.

The Autoregressive (AR) lags order can be mathematically represented as:

$$\varepsilon_{t}:\;AR(p)$$

$$r_{t}=\sum_{i=1}^{p}\delta_{i}r_{t-i}+\varepsilon_{t},$$

$$\left\{\begin{array}{c} \epsilon_{t}∼(WN)\\ \epsilon_{t}∼IID(0,\sigma^{2} \end{array}\right\}$$

Whereas the Moving Average (MA) lags order can be mathematically represented as:

$$\varepsilon_{t}:\;MA(q)$$

$$r_{t}=\sum_{j=1}^{q}\varphi_{j}\varepsilon_{t-j}+\varepsilon_{t},$$

The Moving Average MA(q) is a process in stationary form whereas *ε*_*t*_ is a mean zero with variance *σ*^2^ sole distribution. Therefor ARMA (p, q) is illustrated as follows:

$$r_{t}=\mu +\sum_{i=1}^{p}\delta_{i}r_{t-i}+\sum_{j=1}^{q}\varphi_{j}\varepsilon_{t-j}+\varepsilon_{t}$$

After the proportionate Autoregressive (AR) and the Moving Average (MA) terms of the tentative ARMA models were tested, the information criteria used in selection of the best fits in this study are Akaike Information Criteria (AIC) and Bayesian Information Criteria (BIC).

AIC = −ln(*L*)+2*k*

BIC = −2 ln(*L*)+*k* ln(*T*)

L = the value of the maximized likelihood function

k = *k* = *p*+*q*+1, the constant plus the other parameters.

The model with the lower information criteria value is the better model, meaning the model with lower errors.

After selection of the appropriate ARMA models for the series in study, the next step taken is testing for the existence of serial correlation in the volatility of the series, the ARCH effect, before using the ARMA model as mean equation for the GARCH models.

Omitting the ARCH effects may result in huge losses in asymptotic efficiency (Engle, 1982a) and may lead to excess rejection of the autocorrelation (AC) of normal standardized tests in the conditional mean [26, 27].

The null hypothesis of the ARCH-LM is the absence of ARCH effect in the auxiliary regression it is estimated from, therefore the alternative hypothesis is that, there is ARCH effect in the residuals obtained. The regression is mathematically computed as follows:

$$\epsilon_{t}^{2}=\beta_{0}+\left(\sum_{i=1}^{q}\beta_{1}\epsilon_{t-1}^{2}\right)+v_{t}$$

The squared residuals “$\epsilon_{t}^{2}$” with a an intercept and the total of lag of squared residuals of the order q. The F-statistics, thus the significance of the lagged squared residuals of the neglected variable tests and the [28] ‘s maximum likelihood statistic*R-squared statistics are the outputs from the regression. The ARCH-LM test can be mathematically also represented as:

$${ARCH}_{LM}(q)=TR^{2}$$

Where the total observation is denoted by T and *R*^2^ is the squared residuals.

The ARCH-LM test is used in the post estimation tests to check if the estimated model was able to capture the ARCH effects or not, where rejecting the null hypothesis means that there are still ARCH effects present, and the null hypothesis not rejected means there isn’t ARCH effects left in the time series.

After the autoregressive moving average model (ARMA) is assumed to have ARCH effects, the model is now computed into generalized autoregressive conditional heteroscedasticity (GARCH) model.

In this situation, the model (GARCH (p,q)) is expressed as follows;

The order of GARCH terms *σ*^2^, is represented by p

The order of ARCH terms ∈^2^, is represented by q.

The GARCH (p, q) model is illustrated as follows;

$$yt={x_{t}}^{\prime }b+\in_{t}$$

$$\in_{t}/\varphi_{t-1}∼N(0,\sigma_{t}^{2}$$

$$\sigma_{t}^{2}=w_{0}+\sum_{i=1}^{q}\alpha_{i}\epsilon_{t-i}^{2}+\sum_{j=1}^{p}\beta_{i}\sigma_{t-j}^{2}$$

The specification of the GARCH(p,q) model generally follows these three steps.

Firstly, estimating the best-fitting autoregressive(ARMA) model, then the autocorrelations of the error term is calculated, the last step is testing for significance.

## 4. Empirical results

### 4.1 Descriptive statistics

The summary statistics of the Bitcoin prices, bitcoin volumes, gold prices and gold volumes are presented in the Table 2 below. All the prices been in US dollars, the minimum of bitcoin prices $197 which is less than the minimum of gold prices but the maximum of bitcoin prices $67456 which is greater than the maximum of gold price $2,051 within the same time frame and also the standard deviation of bitcoin prices 16204 which is also greater than the standard deviation of gold prices 274 shows how volatile the bitcoin prices have been comparing to the gold prices within the same period of time. As the skewness of a normally distributed series is zero and the kurtosis of a standard normal distribution is 3, it is obvious that all the series are not normally distributed with gold volume having the highest skewness followed by bitcoin volume, bitcoin prices then gold prices.

**Table 2:** Descriptive statistics for the returns of bitcoin and gold prices and volumes.

Statistic	Bitcoin prices	Gold prices	Bitcoin volume	Gold volume
** Observations**	2087	2087	2087	2087
**Mean**	13002.56	1457.839	1.73E+10	5518.002
** Median**	7215.690	1320.500	8.22E+09	173.0000
** Maximum**	67456.20	2051.500	3.51E+11	386334.0
** Minimum**	197.7020	1055.400	7845880.	1.000000
** Std. Dev.**	16204.03	274.7106	2.11E+10	30474.27
** Skewness**	1.519386	0.464644	3.007471	7.150539
** Kurtosis**	4.239964	1.642458	33.99347	58.71324

### 4.2 Graphical representation of the series

**Fig 1:**
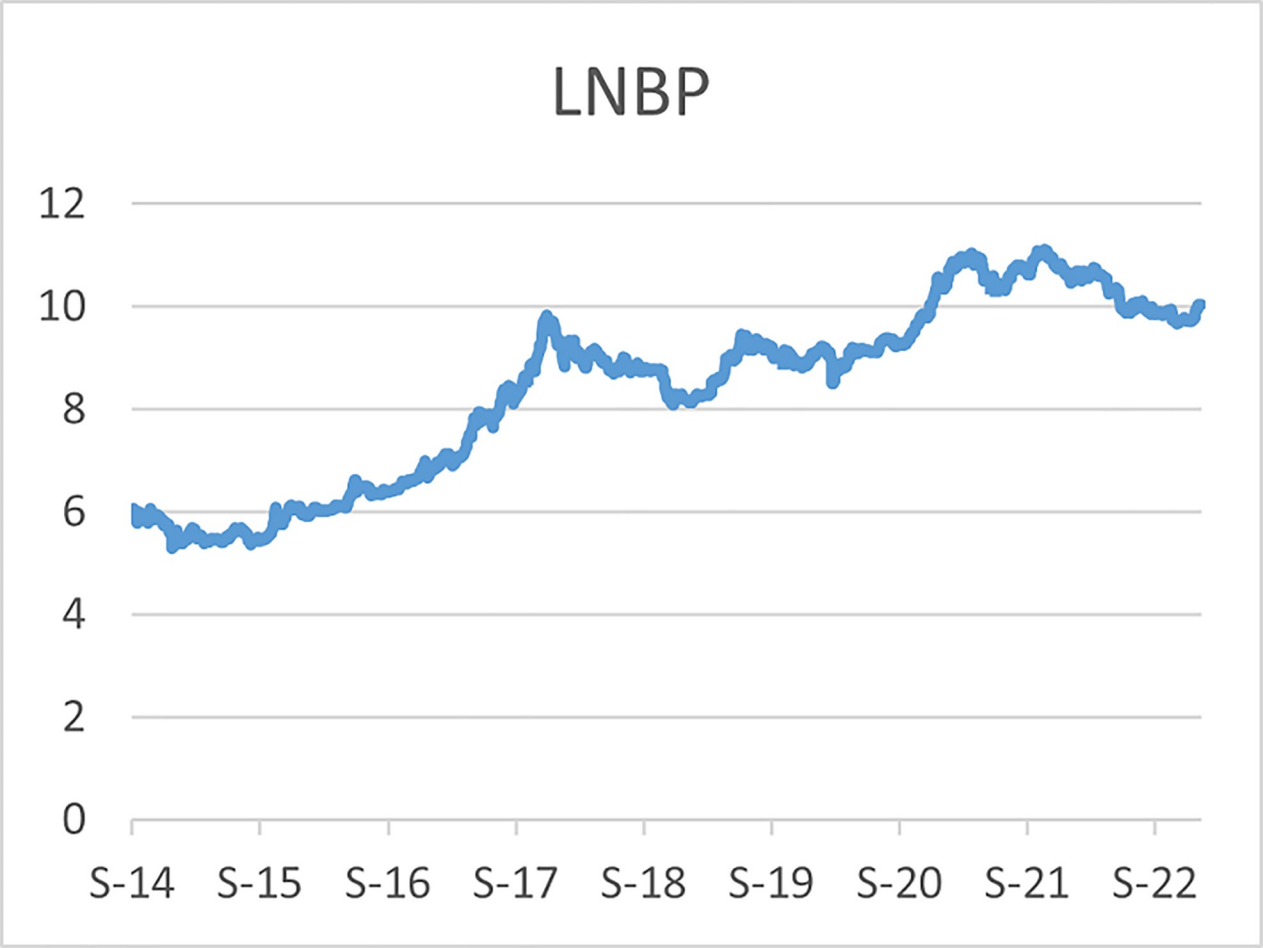
Log of bitcoin daily prices.

**Fig 2:**
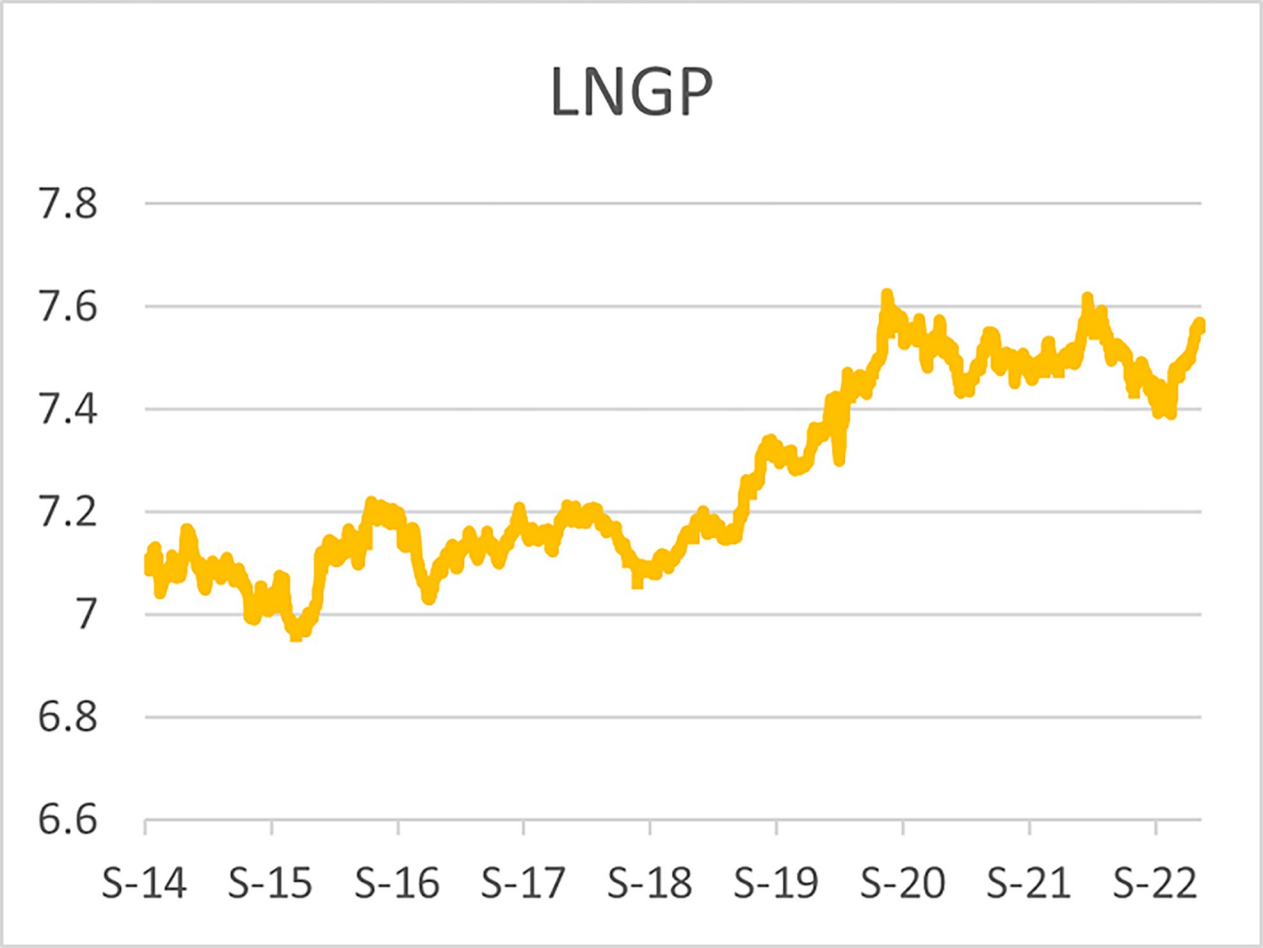
Log of gold daily price.

**Fig 3:**
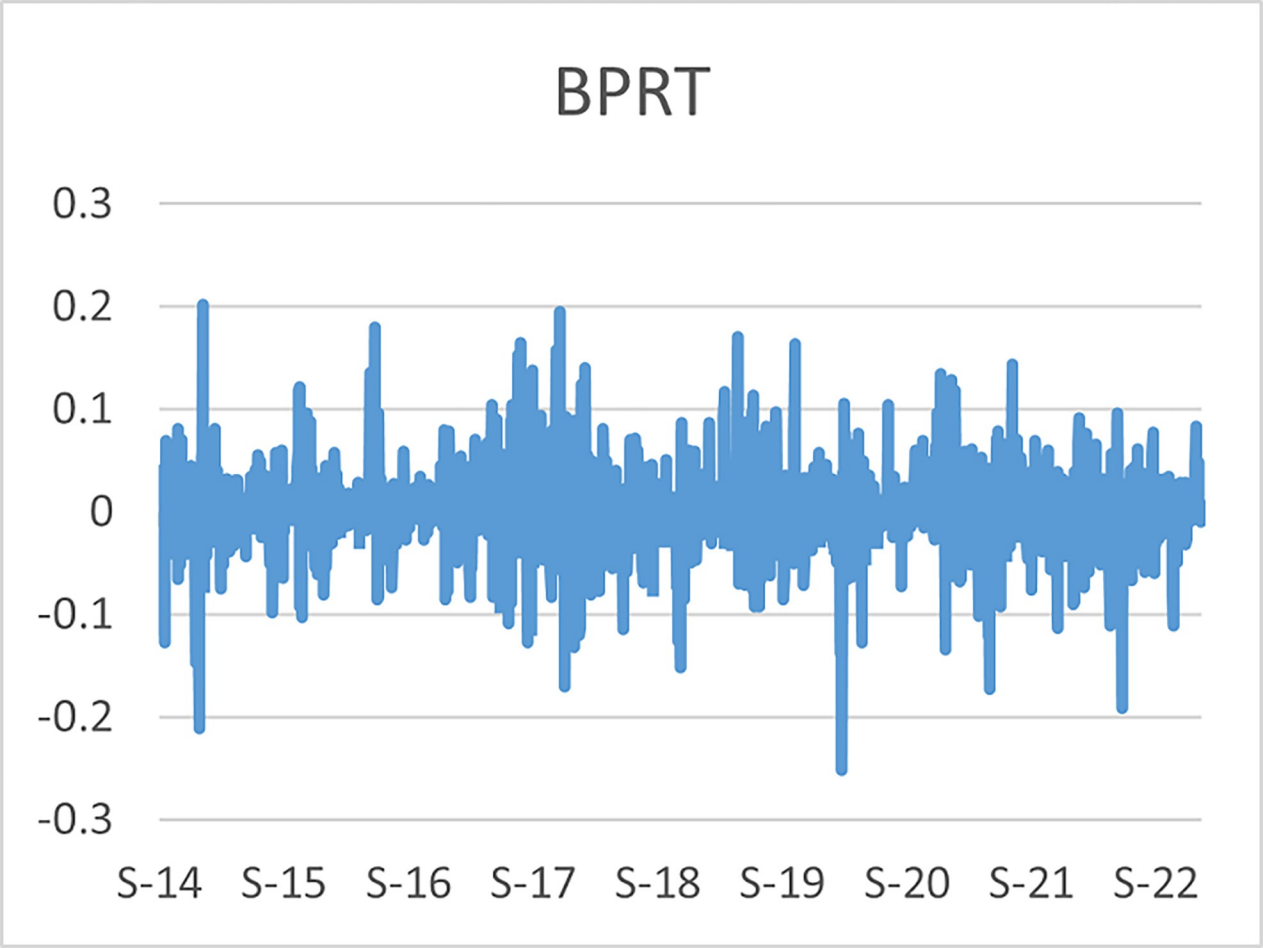
Daily returns of bitcoin prices.

**Fig 4:**
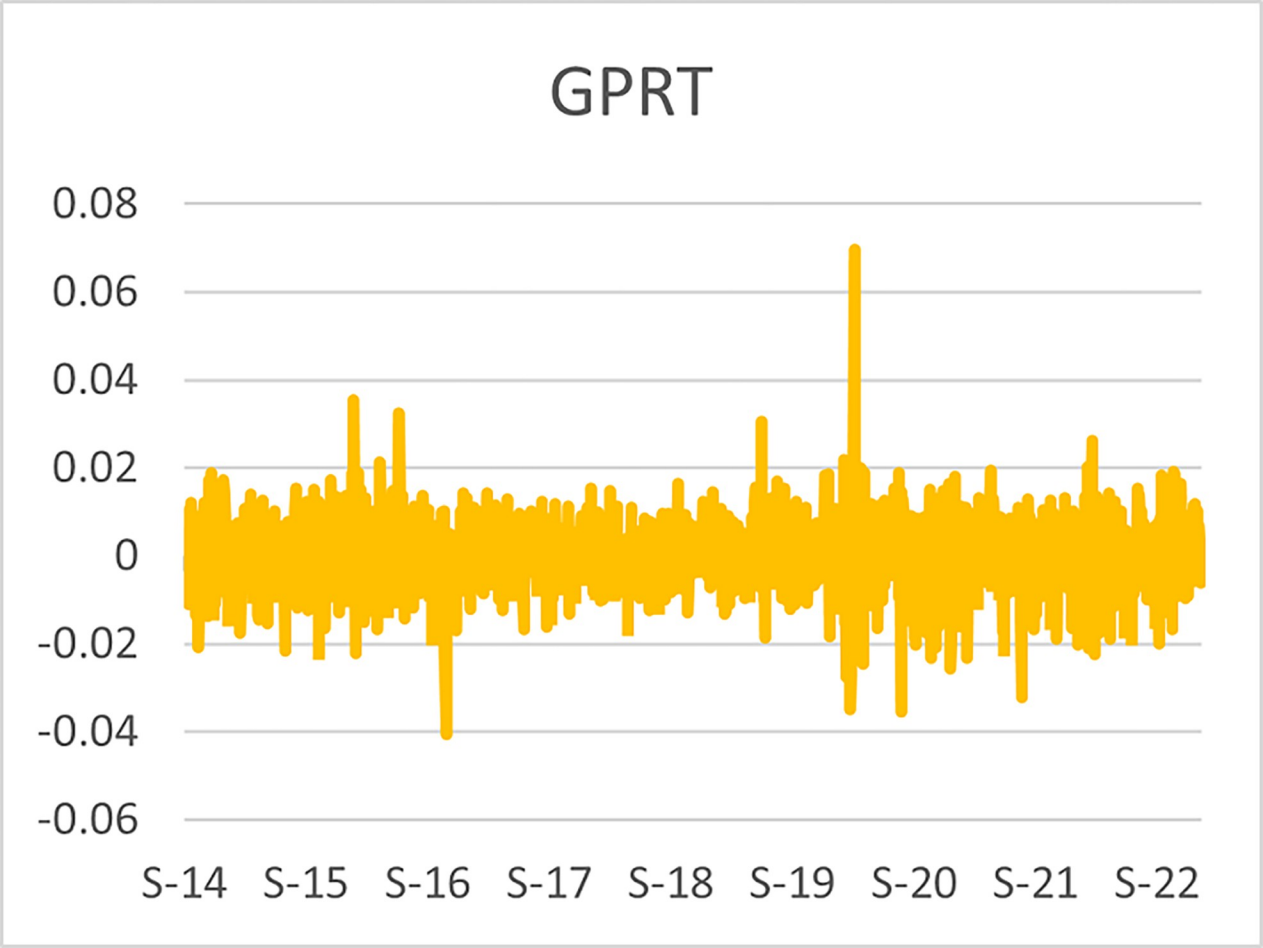
Daily returns of gold prices.

**Fig 5:**
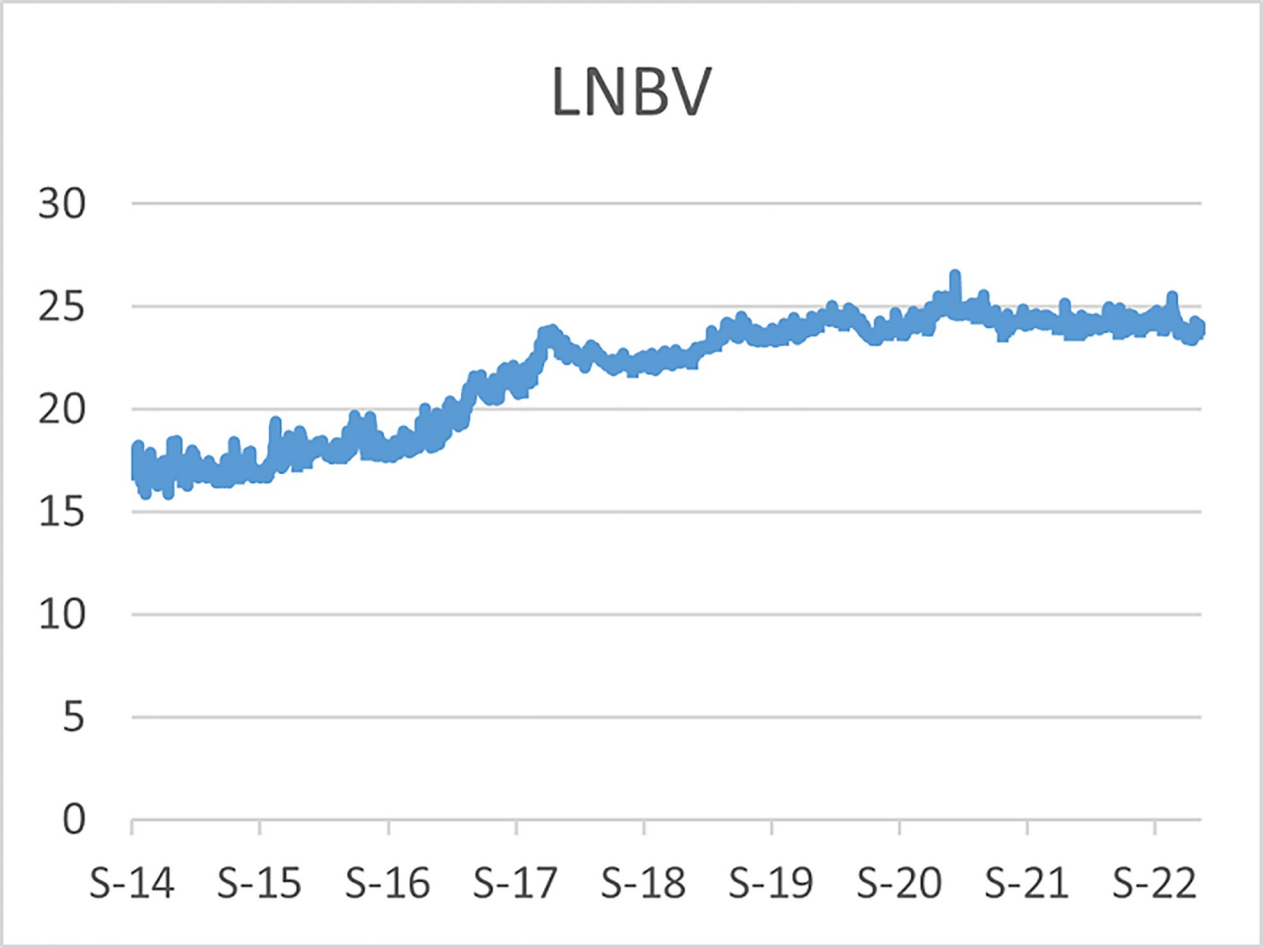
Log of bitcoin daily volume.

**Fig 6:**
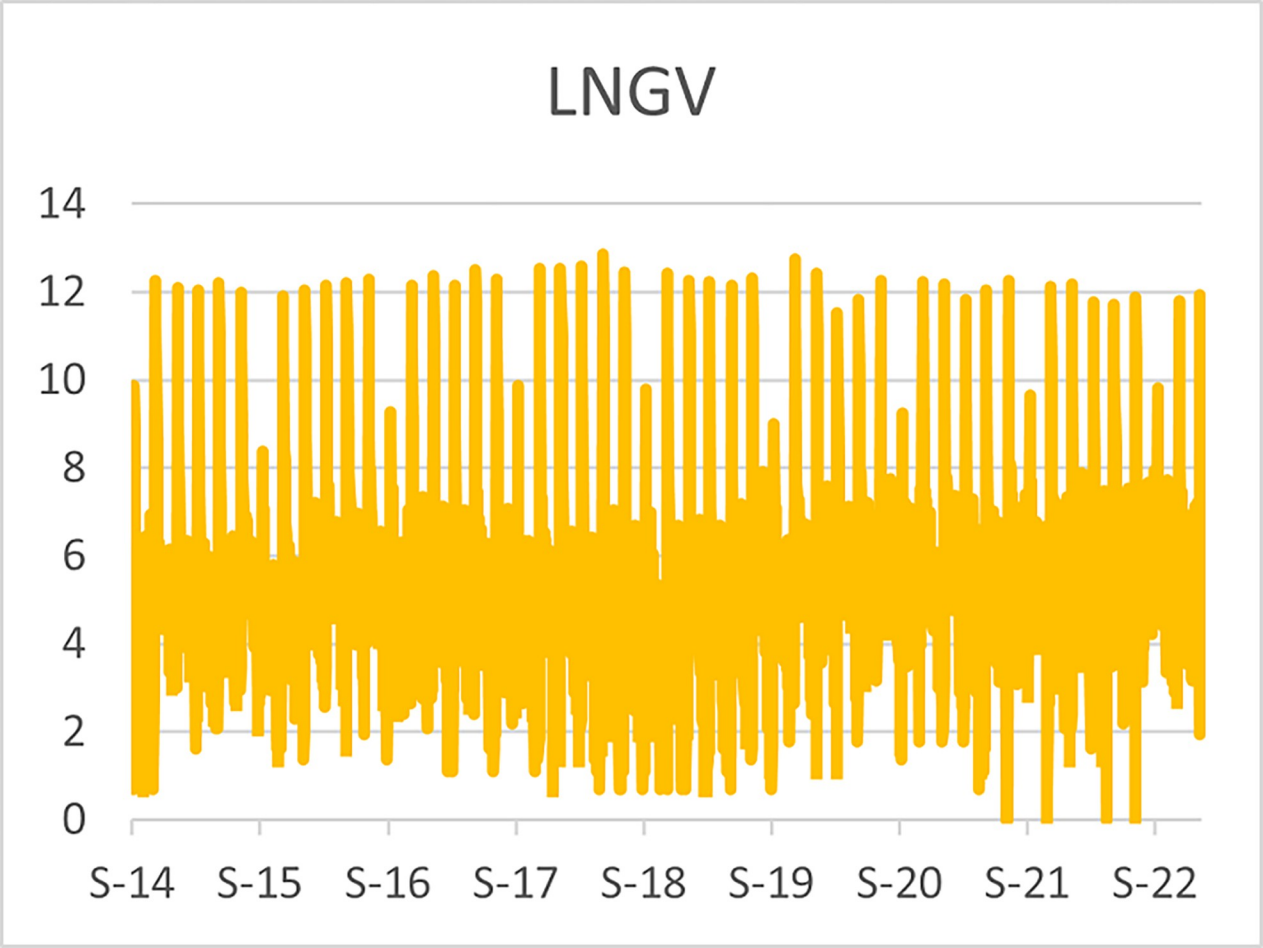
Log of gold daily volumes.

**Fig 7:**
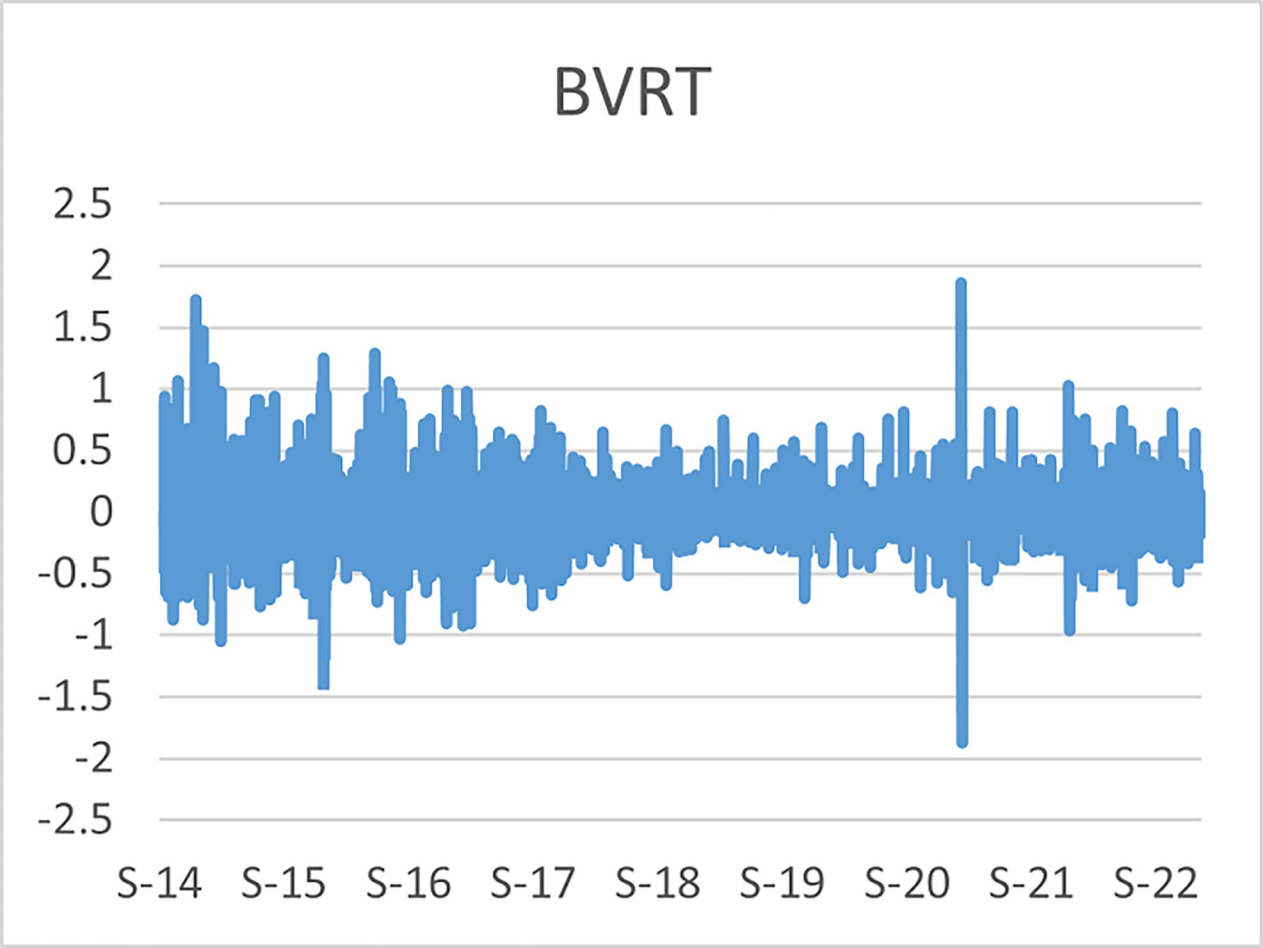
Daily returns of bitcoin volumes.

**Fig 8:**
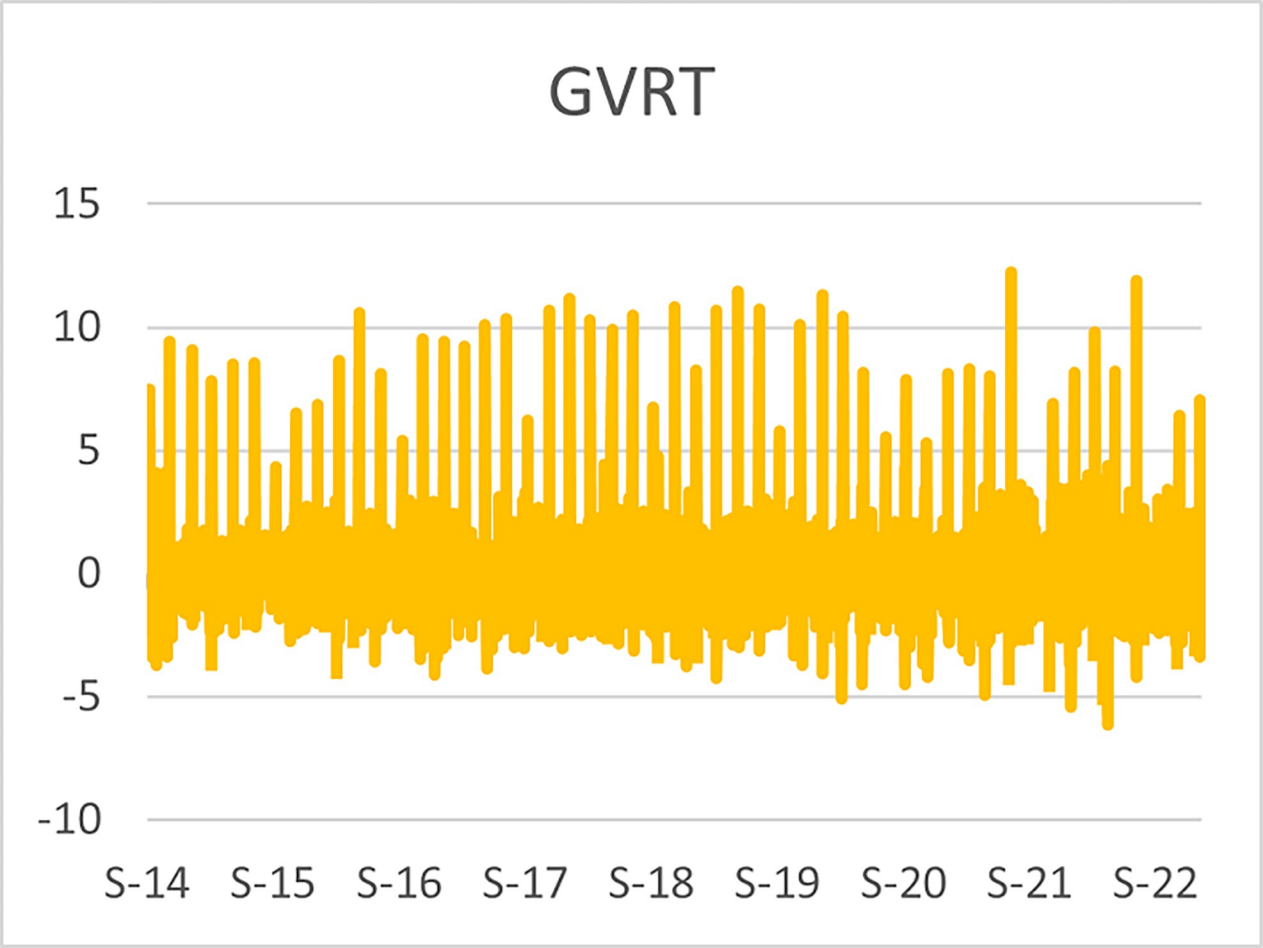
Daily returns of gold volumes.

The original series of daily prices and volumes of bitcoin and Gold as well as the returns as are graphically shown in the graphs below, covering the time period from 22nd September,2014 to 31st Januarry,2023. Natural log of Bitcoin`s daily prices, natural log of Gold`s daily prices are respectively represented by Figs 1 and 2, the daily prices returns are represented by Fig 3 for Bitcoin`s and Fig 4 for Gold`s, Fig 5 visualizes the natural log of bitcoin daily trading volume, Fig 6 for natural log of gold daily trading volume, the daily returns of the volumes are shown in Fig 7 for Bitcoin and Fig 8 for Gold.

In April,2021 the bitcoin market experienced a rapid bull run (rise in price) which took the bitcoin price up to $64,000 then down to $30,000 in June,2021 then a drastic rise again to $67,000 in October,2021, followed by another decline in price in January,2022 and since then the prices declined gradually to $16,000 in the ending of November,2022 and became stable around that prices until early January,2023 where it started to experience bull runs and the prices are around $22,000 in February,2023,before these recent high spikes in prices, the highest prices recorded for bitcoin was in December, 2017 where the bitcoin prices hit $19,000 and declined to $3,300 in December, 2018, thus just a year later. During the recent rise in the bitcoin prices, gold prices faced a slight bear run (fall in prices) from $2,000 in October,2020 to $1,700 and since then the prices declined gradually to $16,000 in the early of November,2022 and it started to another gradual bull runs to around $19,000 as of February,2023.

A similar scenario was also observed during the 2017 rise in price of bitcoin, where gold prices fell from $1300 to $1240 over a period of 4 months before bitcoin hit $19,000. In both scenarios the Gold prices experienced drastic bull runs immediately after bitcoin hits the peaks, now both the bitcoin and gold daily prices are on upward trend, however the volatility of the bitcoins daily prices is too high as compared to the volatility of the golds daily prices.

The daily trading volumes of gold has been almost the same throughout the time period visualized in Fig 6 in logs, while on the other hand the daily trading volume of bitcoin was very low during the early stages as it can be seen in Fig 5 in logs, the bitcoin trading volume begun to rise after the 2017 drastic spikes in its prices and since then it has been on trends with high volatility especially during bull runs, in the recent bull run of the bitcoin prices, its trading volume spiked more than 300 percent and immediately fell back within a day in the ending of March,2021, since has been a downward trend with several upward spikes in the daily trading volume.

The daily price returns of bitcoin and gold is respectively visualized in Figs 1 and 4, both returns show volatility clustering around zero, however the returns of bitcoin prices appear to have higher volatility with leptokurtic nature than the returns of gold with appears to have very intense low volatility around zero as compared to that of bitcoin`s.

The daily trading volumes returns of bitcoin and gold which are respectively graphically shown in Figs 7 and 8 shows vice versa relations that the price returns have. The bitcoin volume returns show clustering around the constant with lower volatility as compared to the volume returns of the gold series where higher volatility around the content zero is visualized.

### 4.3 Unit root test results

Using the Dicky-Fuller, (DF-GLS) Elliott-Rothenberg-Stock Point-Optimal (ERS Optimal) Kwiatkowski-Philips-Schmidt-Shin (KPSS) unit root test methods, the stationarity of the variables was tested firstly at levels and then after their first differenced under two specifications; 1. With intercept only, 2. With both intercept and trend components present.

Below, in Table 3, is the summary of the unit root tests results of the bitcoin price, gold price, bitcoin volume and the gold volume.

**Table 3:** Unit root tests results.

Tests	Variables	At Level		First Difference		Conclusion
		Constant	Trend	Constant	Trend	
Dicky-Fuller GLS (DF-GLS)	LNBP	1.431299	-1.704284	-5.6885***	-10.18773***	I(1)
	LNGP	0.322621	-1.767437	-8.053449***	-30.88029***	I(1)
	LNBV	0.349727	-2.629555*	-1.54987	-3.304861**	I(1)
	LNGV	-5.329354***	-11.36416***	= = =	= = =	I(0)
Elliott-Rothenberg-Stock Point-Optimal (ERS Optimal)	LNBP	115.8036	15.78599	0.062886***	0.129016***	I(1)
	LNGP	25.5858	13.86104	0.033677***	0.102739***	I(1)
	LNBV	56.59193	6.727346*	0.023947***	0.035088***	I(1)
	LNGV	0.171464***	0.221383***	= = =	= = =	I(0)
Kwiatkowski-Philips-Schmidt-Shin (KPSS)	LNBP	5.109178***	0.398272***	0.094558	0.093935	I(1)
	LNGP	4.670181***	0.757744***	0.113589	0.036447	I(1)
	LNBV	5.251569***	0.868175***	0.095321	0.046307	I(1)
	LNGV	0.56292**	0.355199***	0.052664	0.052676	I(0)

One star (*) indicates 10% Significance level, Two stars (**) for 5% and Three stars (***) for 1%

The DF GLS and ELRS optimal unit root tests results for the bitcoin price time series and gold price time series show that the test statistics with constant only and with both constant and trend are statistically insignificant at level but statistically significant at the first difference. That is, the null hypothesis of both the DF GLS test and ELRS optimal unit root tests, which is non-stationarity, cannot be rejected at a level and is rejected after the first difference of the time series at a one percent significant level. These results are confirmed by the KPSS unit root test, which has its null hypothesis as stationarity. The test statistics of the bitcoin and gold prices, while with constant and with both constant and trend, are statistically significant at level but statistically insignificant at the first difference. In other words, they are not stationary at a level but stationary at the first difference. The Bitcoin volume time series proved non-stationary in all the three-unit root tests at the level with only an intercept. With both intercept and trend, the bitcoin volume time series tends to be stationary at the level for DF GLS and ELRS optimal unit root tests but non-stationary for the KPSS unit root test. The bitcoin volume time series becomes stationary for all the three-unit root tests in use with only intercept and with both intercept and trend components present. The gold volume time series is stationary at the level for DF GLS, and ELRS optimal but not for KPSS unit root tests with the presence of only intercept and with both intercept and trend components present.

### 4.4 Identifying the appropriate conditional mean equation

To specify the conditional mean equations for the Garch models used in this paper, the correlogram of the bitcoin price returns, gold price returns, bitcoin volume returns, and the log of gold volume were used in studying the autocorrelation and the partial correlation patterns that each of the variables listed above exhibits. Moreover, the results of the significant tails of the Autoregressive(AR) and Moving Average(MA) were identified as shown in the Table 4 below in the column of the Tentative ARMA models. The process was carried on by using the auto ARMA forecasting method in the EVIEWS with seven lags (as the data series are daily) for both the AR and the MA components. This is to ensure and verify the observations from the correlograms of the variables. The Bayesian Information Criteria (BIC) is used in the selections of the appropriate ARMA models instead of the Akaike Information Criteria (AIC) to avoid over-parameterized models [29].

**Table 4:** Model selection and arch-lm test.

	Tentative ARMA Models		Appropriate ARMA		ARCH EFFECT	
Variables	AR	MA	AR	MA	Without CVD-19	With CVD-19
BPRT	1	1	1	0	82.67199***	81.78807***
GPRT	1,2	1,5	1	0	46.56843***	45.37376***
BVRT	1,2,3,4,5	1,2	1	1	112.3421***	111.2645***
LNGV	1,2,3,4,5,6	1,2,3	5	2	352.4602***	356.3367***

One star (*) indicates 10% Significance level, Two stars (**) for 5% and Three stars (***) for 1%

The heteroscedasticity in the data is then checked by employing the ARCH-LM tests proposed by [28] to check the presences of ARCH effects in the appropriate selected ARMA models. Both the bitcoin and gold price returns follow an AR (1) MA (0) movement, and the bitcoin volume returns follow an AR (1) MA(1) movement, while the movement of the log of gold volumes is AR(5) MA(2). The presence of ARCH effects is checked in two ways: one without the covid-19 component and the other with the covid-19 component. The ARCH effects are significantly found in both scenarios in all the appropriate models selected for all four variables. The table below summarizes the specification of the conditional mean equation.

### 4.5 Garch estimation results

In the estimation of the effects the health crisis (covid-19) has on the volatility of the returns of times series under study, we employed the GARCH model with an ARMA equation as the mean equation, both the bitcoin price returns and the gold price returns mean equations are specified as an AR(1) MA(0) equation, the bitcoin volume returns and log of gold volumes mean equations are specified as an AR(1) MA(1) and AR(5) MA(2) equations respectively.

For each and every time series under study, four(4) different equation specifications are used;

Eq 1 is completely without the covid-19 dummy variable component; the results of the estimation are summarized in the Table 5 below.

**Table 5:** Results of standard garch (p,q) for bprt, gprt, bvrt and lngv, absence of covid-19 variables.

Equations	Parameters	Model 1			
		BPRT	GPRT	BVRT	LNGV
Mean Equation	Constant	0.002658	0.000225	0.004929**	5.315845***
	AR	0.130925***	0.171537***	0.303334***	0.055374***
	MA	---	---	-0.73801***	0.211105***
	Covid-19	---	---	---	---
Variance Equation	Constant	0.000974***	3.80E-05**	0.020569***	0.632956***
	RESID(-1)^2	0.150000***	0.150000***	0.112026***	0.612145***
	GARCH(-1)	0.599733***	0.599973***	0.562026***	0.362774***
	Covid-19				

One star (*) indicates 10% Significance level, Two stars (**) for 5% and Three stars (***) for 1%

Eq 2 has the covid-19 dummy variable present in only the mean equations, the results of the estimation are summarized in the Table 6 below.

**Table 6:** Results of standard garch (p,q) for bprt, gprt, bvrt and lngv, presence of covid-19 variables in mean equation only.

Equations	Parameters	Model 2			
		BPRT	GPRT	BVRT	LNGV
Mean Equation	Constant	0.00247	0.000178	0.005031	5.181236***
	AR	0.263721***	0.229936***	0.308243***	0.049297***
	MA	---	---	-0.742476***	0.203284***
	Covid-19	0.001051	0.000219	-0.004413	0.496084***
Variance Equation	Constant	0.000536***	3.80E-05**	0.058308**	0.641575***
	RESID(-1)^2	0.149563***	0.150000***	0.149770**	0.624228***
	GARCH(-1)	0.599563***	0.600000***	0.599770***	0.353432***
	Covid-19				

One star (*) indicates 10% Significance level, Two stars (**) for 5% and Three stars (***) for 1%

Eq 3 has the covid-19 dummy variable present in only the variance equations, the results of the estimation are summarized in the Table 7 below.

**Table 7:** Results of standard garch (p,q) for bprt, gprt, bvrt and lngv, presence of covid-19 variables in variance equation only.

Equations	Parameters	Model 3			
		BPRT	GPRT	BVRT	LNGV
Mean Equation	Constant	0.002297**	0.000179	0.005817**	5.314962***
	AR	0.279062***	0.226758***	0.280067***	0.053345***
	MA	---	---	-0.714595***	0.207349***
	Covid-19	---	---	---	---
Variance Equation	Constant	6.56E-05***	4.04E-06***	0.003074***	0.532640***
	RESID(-1)^2	0.135588***	0.093283***	0.131840***	0.648463***
	GARCH(-1)	0.822438***	0.824047***	0.828614***	0.345744***
	Covid-19	1.27E-05**	1.77E-06**	0.000138	0.340442***

One star (*) indicates 10% Significance level, Two stars (**) for 5% and Three stars (***) for 1%

Eq 4 has the covid-19 dummy variable present in both the mean equations and the variance equations, the results of the estimation are summarized in the Table 8 below.

**Table 8:** Results of standard garch (p,q) for bprt, gprt, bvrt and lngv, presence of covid-19 variables in both mean equation and variance equation.

Equations	Parameters	Model 4			
		BPRT	GPRT	BVRT	LNGV
Mean Equation	Constant	0.0019	0.000161	0.007197***	5.200896***
	AR	0.278688***	0.226673***	0.281492***	0.048596***
	MA	---	---	-0.716103***	0.200781***
	Covid-19	0.001335	9.34E-05	-0.004499	0.468045***
Variance Equation	Constant	6.54E-05***	4.05E-06***	0.003074***	0.545716***
	RESID(-1)^2	0.135334***	0.093428***	0.132708***	0.652663***
	GARCH(-1)	0.822827***	0.823651***	0.827676***	0.344528***
	Covid-19	1.27E-05**	1.78E-06**	0.000178	0.275265***

One star (*) indicates 10% Significance level, Two stars (**) for 5% and Three stars (***) for 1%

## 5. Discussion of the results of the price returns

The empirical outcomes of the equation without the covid-19 component demonstrate that the influence of past value in predicting current value are greater for returns on gold prices than for returns on bitcoin. The variance in the present value of bitcoin returns constitutes a 0.000974 constant together with a 0.599 (GARCH term) effect from the past variation and a 0.15 component which depends on the past unforeseen errors (ARCH term). This proves to be greater than the gold variance equation, which consists of a 3.80E-05 constant, 0.599 (GARCH term) effects from the past variation, and a 0.15 (ARCH term) component which depends on the past errors. This evidence demonstrate that bitcoin price returns are more volatile than the returns of gold prices. Bitcoin is already very volatile in nature as it is a pure computer-based currency with no guarantee because it is not backed by any legal currency or physical assets, unlike Gold [4, 5]. In other words, bitcoin prices are more volatile than gold prices. The estimation results with covid-19 in the mean equation only show that the covid-19 component is not significant, and the volatility of the bitcoin price returns, and gold price returns exhibit similar movement as in the first equation where the covid-19 component is not included in the equation at all. In equation three, where the covid-19 component is present in only the variance equation, results show that the effects of the covid-19 on the variation of the returns of both bitcoin and gold prices are statistically significant even though the effects are low with 1.27E-05 for bitcoin price returns and 1.77E-05 for gold price returns. However, the presence of the covid-19 dummy variable in the variance equation reduces the ARCH term (the prediction from past errors) from 0.15 (from equation 1 results) to 0.135 for bitcoin price returns and from 0.15 (from equation 1 results) to 0.093 for gold price returns, and it is being captured in the effects coming from the past variation on the present price returns making the increasing the GARCH terms from 0.599 (from equation 1 results) to 0.822 for bitcoin price returns and 0.599 (from equation 1 results) to 0.824 for gold price returns. This clearly shows that the effects of covid-19 on the volatility of bitcoin and gold prices are dependent on the covid-19 component in the variance equation. The effects of the past errors were greater but reduced when the component of the covid-19 was present, [15] argued that an increase in the volatilities of the returns of cryptocurrencies was observed after the announcement of the covid-19 pandemic compared to the periods before the declaration of the covid-19 as a pandemic. Similarly, [16] researched the influence fear sentiments due to the pandemic have on the price dynamics of Bitcoin. They used the hourly Google search data about the covid-19 as a proxy, and their results indicate that the volatility of the Bitcoin market intensively became worse as searches about the covid-19 increased. With the covid-19 component present in both the mean equation and the variance equation, which is just literally the combination of the second and third equations, evidence shows that covid-19 has no significant effects on the mean values of returns of both bitcoin and gold prices but has significant effects on the price return variations. [22] investigated the impacts of the covid-19 on the volatilities of several financial variables, including bitcoin prices and gold prices, by employing the ARMA-EGARCH model using a daily data set from September 2019 to December 2020. They provided evidence that within that short period, the covid-19 pandemic had a statistically significant impact on the bitcoin and gold prices, causing excessive fluctuations in their prices and contaminating mutual volatilities between them, similarly to [30] contaminating spillover effects among cryptocurrency prices during covid-19 crisis. Argued that, its due to their financial imperfection dynamics [31].

### 5.1 Discussion of the results of the bitcoin volume returns and log of gold volumes

The empirical results of the equation without the covid-19 component show that the mean equation of both the bitcoin volume returns and the log of gold volumes consists of statistically significant constants, autoregressive components, and moving average components. In the variance equation, the effects of the past variation (GARCH term) on the present variation of bitcoin volume returns is greater than that of the golds; however, the effects coming from the past errors (which are unexplained) from the log of the gold volumes is greater than that of the bitcoins volumes. With covid-19 present in only the mean equation, the results show that covid-19 has no statistically significant role in the determination of the returns of the bitcoin volume but covid 19 has a statistically significant impact on the determination of the mean values of the log of gold volumes. Evidence from the results of the Garch model in the third equation where covid-19 is present in only the variance equation testifies that covid-19, with the estimation value of 0.340442, proves to be statistically significant for the gold volumes but statistically insignificant for the determination of the variation of the bitcoin volume returns with the estimation value of 0.000138. In other words, covid-19 greatly affects the volatility of gold volumes but does not have any statistically proven effect on the volatility of bitcoin volumes. With the covid-19 component present in both the mean equation and the variance equation, which is just literally the combination of the second and third equations, evidence shows that covid-19 has no significant effects on the mean values of returns of bitcoin volumes but has statistically significant effects on the gold volume mean values and volatility. Similarly, to [9, 17, 32],where they found that the supply of Gold was hugely affected by the covid-19.

## 6. Conclusion

Since the emergence of bitcoin into the global financial markets, numerous studies have been done to examine the connections between the blockchain’s bitcoin and the well-known precious metal, Gold. While investors are deciding on which of them (bitcoin and gold) to dive into and when to invest, knowledge of the similarities and differences between them and the role they play under various circumstances are necessary. The Covid-19 pandemic, although a health crisis, however, in this technologically advanced era, where all sectors of the world economy are more tightly integrated than ever before, it caused several fatalities and restricted the movements of humans around the world. Both the physical and digital financial markets were affected by the shock of the covid-19 pandemic. This paper examines the effects covid-19 has on the volatilities of the dynamics of gold and bitcoin. Gold has a long history of being seen as a safe asset amid financial crises, as it has been seen as a natural currency and a very strong value-storage precious metal. Additionally, it is asserted that it is negatively correlated with financial cycles, which provides shade during a financial crisis [9]. Evidence from [10], due to the low responsiveness of gold supply to price changes, the price of Gold in the short run is mainly determined by the forces coming from demand. In the long run, both the forces of demand and supply set the price of Gold. Monetary macroeconomic instruments and political activity indicators mostly have great effects on the price of Gold. This research makes use of daily data of bitcoin prices and volumes, gold prices and volumes from 9/22/2014 to 1/31/2023, and a dummy variable to represent covid-19, effective from March 11, 2020, to 1/31/2023. The GARCH model is used in this study to examine the effects of the Covid-19 health crisis on the volatility of bitcoin and gold prices and volume. ARMA is used as the mean equation. Bitcoin and Gold are both traded on online platforms. However, bitcoin lacks a physical form because it is digitally created and cannot be utilized in any physical form, but gold has a physical form because it is mined and used in numerous physical ways. These parallels and contrasts help in understanding the findings of this paper. The results from the estimated GARCH models show that during the period of covid-19, which is not a crisis coming from the financial sector of the world nor a cyber-attack into the financial markets, only the price volatilities of bitcoin and gold increased, but no significant impact on their mean values was recorded. This makes much sense as all the functions of both bitcoins, and the digital aspect of Gold could be fully realized during the covid-19 pandemic. However, only the fluctuations in their prices increased as people were in times of distress but not in haste to liquidate their assets. Covid-19 restricted the movement of people all over the world, which had an impact on the mining, transporting, and physical usage of Gold. This finding is in line with [19], they found that after the announcement of the covid-19 as a pandemic, the volatility of the returns of cryptocurrencies became more intense. Similarly, the findings of [20], the influence of fear sentiments due to the pandemic on the price dynamics of Bitcoin, their results also indicate that the volatility of the Bitcoin prices intensively became worse as searches about the covid-19 increased on the internet.

The empirical results of this paper also testify that covid-19 affects both the mean values and the volatilities of the gold volumes but has no effect on both the mean values and volatilities of the bitcoin volumes, that is, the positive trend in the prices of both bitcoin and Gold continued throughout the period of the pandemic, this finding highlights the significant impact the restrictions of physical movements of people during the pandemic had on gold but not on bitcoin, as bitcoin has no physical form. This empirical finding of our study goes with [10], their findings suggest that, due to the supply of Gold being hugely affected by the covid-19, while Gold has the capability of providing safe heaven during crisis, the increase in demand with the decrease in supply results in a rise in prices during the pandemic.

The results from the study simply highlight that covid-19 pandemic had no significant negative impact on the prices of bitcoin and Gold, but the impact on their respective volumes were different. The volatility of the Gold volumes were significantly affected, however, the volumes of bitcoin were not touched by the covid-19 pandemic. This shows how significant the role of the differences (bitcoin being fully digitally produced with no physical form compared to gold) and similarities (both assets having digital trading platforms), play when facing an externality like the covid-19 pandemic. Further research can be conducted with different estimation methods directed to the period of time it may take for the shocks of the pandemic to fully die from the bitcoin and gold markets and the possible aftermaths. Also, this area of study can be broadened with further research directed to different financial assets with similar characteristics like other cryptocurrencies or precious metals, or companies in the stock markets to understand how they perform during an external crisis like the covid-19 pandemic, wars or critical political changes in countries with large open economies.

## Supporting information

S1 File (XLSX)
